# New Appliances and Things Medical

**Published:** 1900-08-04

**Authors:** 


### NEW APPLIANCES AND THINGS MEDICAL.

**[We shall be glad to receive, at our Office, 28 & 29, Southampton Street, Strand, London, W.C., from the manufacturers, specimens of all new preparations and appliances which may be brought out from time to time.]**

### SHREDDED WHEAT.

(The Shredded Wheat Co., 678, Eastcheap, E.C.)

Shredded wheat is a very interesting substance. How far it will be a success, how far, that is, it will take the fancy of the people and become a general article of diet we cannot say. Much will of necessity depend upon the cooks and upon whether they will take the trouble to make it into tasty and palatable dishes, but so far as the stuff itself is concerned we can have no hesitation in expressing a very favourable opinion, both in regard to its nutritive power and its digestibility. We need not give the analysis. Practically shredded wheat contains the same substances, and in nearly the same proportions, as wheat itself. Indeed, considering the method of its manufacture, it could not be otherwise, and thus, so far as whole wheat can be regarded as a complete food, the shredded wheat may claim the same description. The object of the manufacturers seems to have been to produce a cooked substance containing the whole of the wheat grain, and to turn it out in such a form that no "raising" or fermenting process shall be required to separate the particles grain from grain, as must always be the case with every kind of flour to enable the salivary and other digestive juices to permeate the mass when it is eaten. From the early dawn of civilisation people have endeavoured to render wheat palatable and digestible. Most of the methods used have consisted in first reducing the grain to a fine flour, then into a paste, and then by varions processes, generally involving the use of some ferment or other, turning this paste into a spongy substance, such as bread, of a sufficiently open texture to allow the digestive juices to pass throughout its substance. The inventor of shredded wheat has aimed at producing an open textured substance—shredded wheat—direct from the grain, without any preliminary grinding into flour, or making into paste or dough, and without the employment of any leaven or ferment or other aërating process. This he has accomplished by first cleaning and boiling the wheat, and then passing the softened grain between grooved rollers. The result is that all the various constituents of the wheat are thoroughly mixed and appear in the form of long fine shreds, each thin enough to be dealt with by the saliva, and each firm enough to prevent its falling into a squashy mass when wet. The shreds are much thinner than the fibres of vermicelli, and each contains a mixture of all parts of the wheat. These shreds then form the basis of shredded wheat, which comes out from the oven in form of light porous biscuits entirely formed of these firm, hard, dry fibres, separated by abundant air spaces, by aid of which every shred is capable of being reached by the digestive juices. It must be confessed that the material has not a very attractive appearance, and that on first putting some of it into one's mouth the sensation is that of having got hold of a very dry morsel indeed. A very little mastication, however, brings out its flavour, and shows its extraordinary power of setting up a flow of saliva, by which sweetness is rapidly developed. To make it nice from the start is merely a matter of cooking and preparation. Personally, we soon found it pleasant to eat as a biscuit, even dry in its native state, and we have no doubt that it will be found a useful basis for many dishes. Of its purity, its nutritious qualities, and its digestibility there can be no question.

